# Behavioral and transcriptomic analysis of Trem2-null mice: not all knockout mice are created equal

**DOI:** 10.1093/hmg/ddx366

**Published:** 2017-10-11

**Authors:** Silvia S Kang, Aishe Kurti, Kelsey E Baker, Chia-Chen Liu, Marco Colonna, Jason D Ulrich, David M Holtzman, Guojun Bu, John D Fryer

**Affiliations:** 1 Department of Neuroscience, Mayo Clinic, Jacksonville, FL 32224, USA; 2 Department of Pathology and Immunology, Washington University, St. Louis, MO 63110, USA; 3 Department of Neurology, Washington University, St. Louis, MO 63110, USA; 4 Neurobiology of Disease Graduate Program, Mayo Clinic Graduate School of Biomedical Sciences, Jacksonville, FL 32224, USA

## Abstract

It is clear that innate immune system status is altered in numerous neurodegenerative diseases. Human genetic studies have demonstrated that triggering receptor expressed in myeloid cells 2 (*TREM2*) coding variants have a strong association with Alzheimer’s disease (AD) and other neurodegenerative diseases. To more thoroughly understand the impact of TREM2 *in vivo*, we studied the behavioral and cognitive functions of wild-type (WT) and *Trem2^−/−^* (KO) mice during basal conditions and brain function in the context of innate immune stimulation with peripherally administered lipopolysaccharide (LPS). Early markers of neuroinflammation preceded *Aif1* and *Trem2* upregulation that occurred at later stages (24–48 h post-LPS). We performed a transcriptomic study of these cohorts and found numerous transcripts and pathways that were altered in *Trem2^−/−^* mice both at baseline and 48 h after LPS challenge. Importantly, our transcriptome analysis revealed that our *Trem2^−/−^* mouse line (Velocigene allele) results in exaggerated *Treml1* upregulation. In contrast, aberrantly high *Treml1* expression was absent in the *Trem2* knockout line generated by the Colonna lab and the Jackson Labs CRISPR/Cas9 *Trem2* knockout line. Notably, removal of the floxed *neomycin* selection cassette ameliorated aberrant *Treml1* expression, validating the artifactual nature of *Treml1* expression in the original *Trem2^−/−^* Velocigene line. Clearly further studies are needed to decipher whether the *Treml1* transcriptional artifact is functionally meaningful, but our data indicate that caution is warranted when interpreting functional studies with this particular line. Additionally, our results indicate that other Velocigene alleles or targeting strategies with strong heterologous promoters need to carefully consider downstream genes.

## Introduction

Microglia are the central nervous system (CNS) resident innate immune cell population that act as sentinels and continually sample and survey the local milieu in the brain parenchyma (1–4). Microglia participate in both basal and pathological disease states, orchestrating aspects of processes including development, synaptogenesis, and inflammation (5,6). Notably, microglial reactivity has been demonstrated in numerous neurodegenerative disorders, including Alzheimer’s disease (AD), frontotemporal dementia (FTD), and Parkinson’s disease (PD), suggesting that they may play a pivotal role in the outcome of disease progression (7–13). Within the context of AD, many of the human genetic risk factors, including *APOE*, *CLU*, *CD33*, and *TREM2*, are genes that have the potential to alter innate immunity, making it critical to understand how these factors regulate microglial function (14–20).

Triggering receptor expressed on myeloid cell 2 (TREM2) is an immunoglobulin (Ig) superfamily cell surface receptor that is expressed by macrophages, dendritic cells, and osteoclasts in the periphery (21–24). Within the CNS, TREM2 is expressed almost exclusively on microglia and is one of the more highly expressed molecules that constitute the microglial sensome (25–27). TREM2 engagement results in intracellular signaling mediated by its association with TYRO protein tyrosine kinase binding protein (TYROBP), also known as DNAX-activating protein of 12 kDa (DAP12) (22,28,29). Several ligands for TREM2 have been revealed including lipopolysaccharide (LPS), bacteria, anionic and zwitterionic lipids, and, interestingly, both apoE and CLU have been identified as TREM2 ligands, potentially converging several human AD risk gene pathways together (30–34). The R47H allele as well as other TREM2 variants have been associated with increased risk of AD as well as FTD and PD (18–20, 35,36). Additionally, *TREM2* or *TYROBP* loss of function mutations result in Nasu-Hakola disease (NHD), also known as polycystic lipomembranous osteodysplasia with sclerosing leukoencephalopathy (PLOSL), characterized by microglial activation and dementia. These human genetic data indicate a critical role for TREM2 in neuropathological outcomes (18–20,37,38). TREM2 deficiency is associated with increased pro-inflammatory cytokine production following either *in vitro* and *in vivo* LPS stimulation and decreased bacterial clearance, suggesting an anti-inflammatory, protective function (24,39,40). Additionally, TREM2 has been implicated in shaping microglial phagocytosis of apoptotic neurons, microglial clustering around amyloid plaques, and microglial expansion and survival (34,41–46). Although data has emerged focusing on TREM2 during disease, it remains unclear whether a TREM2 deficiency actually impacts basal behavioral and cognitive function. Moreover, it is unknown how TREM2 impacts CNS transcriptomes under homeostatic conditions following systemic inflammation.

Here, we used a *Trem2* knockout mouse model generated from the Velocigene ‘definitive null’ targeting strategy in which the entire coding region was replaced by selection cassette (lacZ-flox-human Ubiquitin C promoter-neomycin-flox), beginning at 16 bp upstream of the ATG start codon and ending at the TGA stop codon of *Trem2*. We demonstrate that TREM2 has no impact on behavioral and cognitive readouts as well as little to no impact on early CNS pro-inflammatory cytokine levels following peripheral LPS challenge, suggesting that TREM2 may be dispensable under basal conditions and at early time points after systemic inflammation. Following peripheral LPS challenge, we show that TREM2 is not significantly up-regulated until 48–72 h post challenge, well past the point of the initial wave of inflammation. Examination of the transcriptomes from WT and *Trem2^−^^/^^−^* mice revealed alterations in several genes 48 h after LPS challenge; however, the predominant transcript that was overwhelmingly elevated in *Trem2^−^^/^^−^* mice, *Treml1*, was also observed under basal conditions. Examination of *Treml1* expression in the *Trem2^−^^/^^−^* line generated in the Colonna lab (24) revealed a trend for a slight increase in expression and a recently generated *Trem2* CRISPR/Cas9 knockout allele had no significant change in *Treml1* expression. Removal of the floxed neomycin cassette, driven by the human ubiquitin C promoter, abolished the *Treml1* overexpression artifact in the Velocigene knockout line. These data suggest that the original Velocigene targeting of the *Trem2* locus introduced an artifactual alteration in *Treml1* expression that is of unknown consequence and caution must be taken while interpreting the role of *Trem2* with this particular mouse model if the neomycin cassette remains intact.

## Results

### *Trem2* deficiency does not alter behavior or cognition during basal conditions

**Figure 1.**
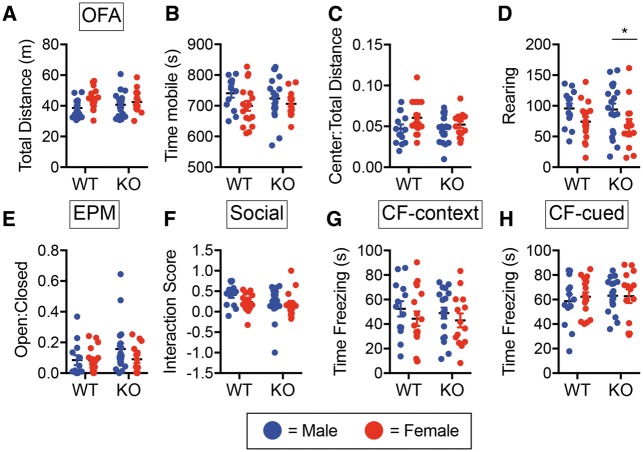
*Trem2* deficiency does not alter behavioral or cognitive performance. Six-month-old male and female C57BL/6 N wild type (WT) or *Trem2^−/−^* (KO) mice were examined for behavioral alterations in the open field assay for mobility and anxiety (**A–D**), elevated plus maze for anxiety (**E**), and the 3 chamber social test of sociability (**F**). Contextual (**G**) and cued (**H**) cognitive memory performance was determined using conditioned fear assays. Males (blue) and females (red) had similar results. Shown are the averages ± S.E.M. for *N* = 13–19 animals/group/sex (WT female *N* = 17, WT male *N* = 13, KO female *N* = 14, KO male *N* = 19). There were no significant genotypes or sex by genotype differences noted in any of the assays (Two-way ANOVA, Fisher’s LSD).

To determine if TREM2 deficiency has an impact on behavioral or cognitive abilities under basal conditions, 6-month-old male and female WT and *Trem2^−^^/^^−^* mice from the Velocigene allele were run through a series of behavioral and cognitive tests. Open field assay (OFA) was used to examine general activity/locomotion and anxiety but revealed no significant differences between the two genotypes in mobility (total distance traveled, time mobile) (Fig. 1A and B) or anxiety (center: total distance ratio, rearing) (Fig. 1C and D). Although there was a slight decrease in rearing between female versus male *Trem2^−^^/^^−^* mice, there were no significant genotype by sex effects. Use of the elevated plus maze (EPM) as another anxiety readout also showed no difference between WT and *Trem2^−^^/^^−^* mice in open: closed arm ratios (Fig. 1E), indicative of similar overall anxiety-like behavior. Additionally, three chamber social interaction behavioral testing to examine sociability in WT and *Trem2^−^^/^^−^* revealed comparable interaction scores (Fig. 1F). We next used conditioned fear (CF) behavioral testing to assess whether there were any alterations in cognitive function between 6-month old WT and *Trem2^−^^/^^−^* mice. Similar memory retention observed between WT and *Trem2^−^^/^^−^* mice in both the hippocampus-dependent context test and the hippocampus/amygdala-dependent cued test (Fig. 1G and H). Together these data show that *Trem2* deficiency has no overt impact on behavioral and cognitive parameters at 6-months of age. Since there were no sex by genotype effects, the remainder of the study was conducted with male mice.

### *TREM2* does not impact early CNS inflammation after systemic LPS challenge

**Figure 2.**
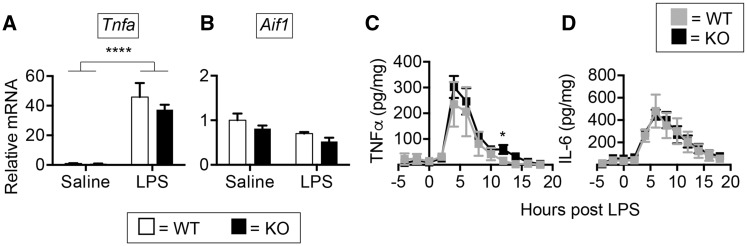
TREM2 does not impact early CNS inflammation following a peripheral inflammatory challenge. Hippocampal tissues from PBS perfused male brains were isolated from C57BL/6 N wild type (WT) or *Trem2^−/−^* (KO) mice 4 h post i.p. challenge with 2 mg/kg LPS. RT-qPCR was conducted for (**A**) *Tnfa* and (**B**) *Aif1* (encodes IBA1) levels. Shown are the averages ± S.E.M. for *N* = 4–7 mice per group. No significant differences based on genotype were noted. A main effect of LPS was noted for *Tnfa*, but no *Trem2* genotype effect was observed (Two-way ANOVA, Tukey post-hoc, **P ≤ 0.01, ***P≤ 0.001). Hippocampal interstitial fluid (ISF) was collected from WT and *Trem2^−/−^* mice 4 h prior to and 18 h post i.p. injection with 2 mg/kg LPS using *in vivo* microdialysis. ISF fractions pooled in 2-h increments were measured for (**C**) TNFα and (D) IL-6. No significant *Trem2* genotype differences were found with the exception of slight elevation of TNFα at the 12 h time point (Multiple *t*-test, Benjamini and Hochberg post hoc, *P ≤ 0.05). Shown are the averages ± S.E.M. for *N* = 3 mice per group.

TREM2 has been shown to modulate pro-inflammatory cytokine production by microglia and macrophages after *in vitro* challenge with the gram negative bacterial mimetic, lipopolysaccharide (LPS) (24,40). Although *Trem2* deficiency had no overt impact on behavioral/cognitive parameters during homeostatic conditions, it is possible that its effect is only revealed in adult animals within the context of inflammation. To examine if *Trem2* expression impacts early CNS inflammation associated with systemic LPS challenge, WT and *Trem2^−^^/^^−^* mice were injected with 2 mg/kg LPS i.p. and PBS perfused brains were harvested and dissected 4 h after injection. RT-qPCR revealed a significant increase in hippocampal *Tnfa* transcript levels in both LPS challenged WT and *Trem2^−^^/^^−^* mice, but no effect of genotype on the magnitude of the response (Fig. 2A). Additionally, there was no difference in the expression of *Aif1* (encoding the microglial marker IBA1) between WT and *Trem2^−^^/^^−^* mice, suggesting a lack of significant microglial activation at this time point for both genotypes (Fig. 2B). To further address any early neuroinflammatory changes that may occur in the absence of *Trem2*, *in vivo* microdialysis was conducted to monitor TNFα and IL-6 levels in the hippocampal interstitial fluid prior to and up to 12 h after peripheral LPS injection. Quantitative cytometric bead array analysis of TNFα levels revealed no significant differences in either basal levels (−4, −2, and 0 h before LPS injection) or early induction of TNFα protein in the CNS interstitial fluid of WT (grey line) or *Trem2^−^^/^^−^* (black line) mice (Fig. 2C). Between 12 and 14 h post challenge, there was a small but significant difference between WT and *Trem2^−^^/^^−^* TNFα levels in the hippocampus. No significant alterations based on genotype were observed in IL-6 levels at any of the times examined (Fig. 2D). These data suggested that TREM2 does not impact early CNS inflammation that is observed following systemic inflammation.

### Delayed microglial activation and CNS *Trem2* induction after LPS challenge

**Figure 3.**
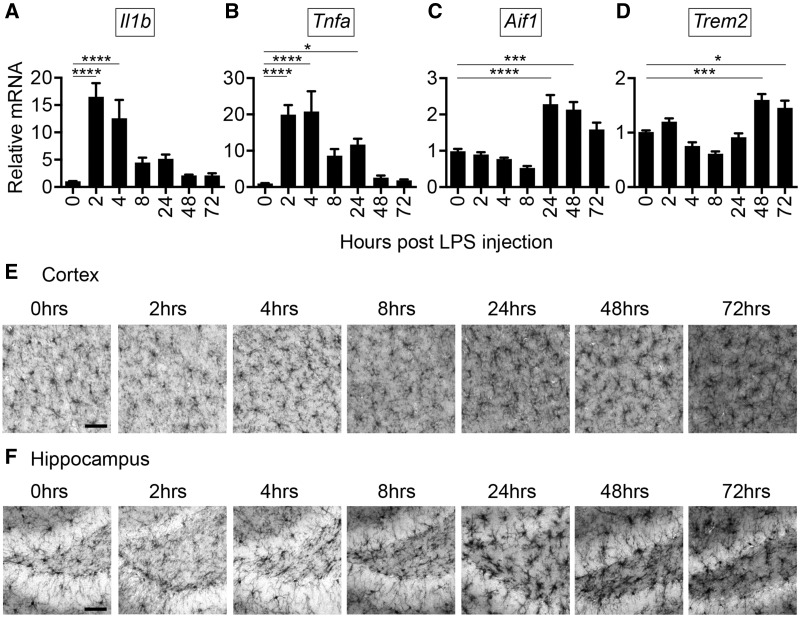
Systemic inflammation induces CNS inflammation that precedes robust microglial activation and *Trem2* induction. PBS perfused hemi brains from male C57BL/6 N mice were harvested at 0, 2, 4, 8, 24, 48, and 72 h post i.p. injection with 2 mg/kg LPS. RT-qPCR was conducted for (**A**) *Il1b*, (**B**) *Tnfa*, (**C**) *Aif1* (encodes IBA1), and (**D**) *Trem2*. *Il1b* and *Tnfa* induction preceded *Aif1* and *Trem2* upregulation that was noted after the majority of inflammation subsided. Shown are the averages ± S.E.M. for *N* = 4–7 mice per group, *N* = 2 independent experiments. (One-way ANOVA, Tukey post-hoc, *P ≤ 0.05, ***P≤ 0.001, ****P ≤ 0.0001). IBA1 immunohistology was conducted on 50 μm sections from male mice harvested at 0, 2, 4, 8, 24, 48, and 72 h post i.p. injection with 2 mg/kg LPS and examined for reactivity in the (**E**) cortex and (**F**) hippocampus. Significant alterations in IBA1 reactivity was noted starting at 24 h post injection. Shown are representative images from *N* = 6–8 mice from *N* = 2 independent experiments. Scale bar = 50 μm.

Although early TNFα production was evident, the absence of *Aif1* induction at 4 h suggested that early time points might not address alterations in microglial function that could occur with *Trem2* deficiency. To determine how CNS microglial activation is coordinated with inflammation and *Trem2* expression following systemic LPS challenge, WT mice were injected with 2 mg/kg of LPS intraperitoneally (i.p.) and PBS perfused brains were harvested at 0, 2, 4, 8, 24, 48, and 72 h post injection. RT-qPCR demonstrated an early elevation in the expression of *Il1b* and *Tnfa* pro-inflammatory genes at 2 and 4 h followed by a drop in expression (Fig. 3A and B). Interestingly, *Aif1* was not elevated during the early stage of robust inflammation (2 and 4 h post injection) and had a delayed induction observed at later time points (24 and 48 h post injection) (Fig. 3C). *Tnfa* levels were slightly but significantly still elevated at 24 h when increases in *Aif1* were observed. Notably, *Aif1* induction preceded induction of *Trem2* gene expression, which was observed at 48 and 72 h post injection (Fig. 3D). IBA1 immunohistochemistry supported the delayed microglial activation identified by RT-qPCR after peripheral LPS stimulation, with microglia that appeared to look more activated after 24 h post challenge (Fig. 3E and F). The kinetics of altered IBA1 immunoreactivity was similar in both the cortex and hippocampus regions of the brain (Fig. 3E and F). Delayed *Trem2* upregulation is suggestive of a potential anti-inflammatory role following systemic inflammation.

### *Trem2* mildly represses CNS inflammation under both basal and LPS-stimulated conditions

TREM2 has been suggested to impact several different aspects of microglial function including phagocytosis, inflammation, and survival (24,39–42,44,45). In order to examine the impact of TREM2 on CNS responses following systemic inflammation using an unbiased approach, RNAseq was conducted on hippocampal RNA isolated from PBS perfused saline or LPS injected WT or *Trem2^−^^/^^−^* mice 48 h post challenge when *Trem2* is upregulated (Fig. 3D). RNAseq transcriptome data were analysed with false discovery rate (FDR) set at 5%.

**Figure 4.**
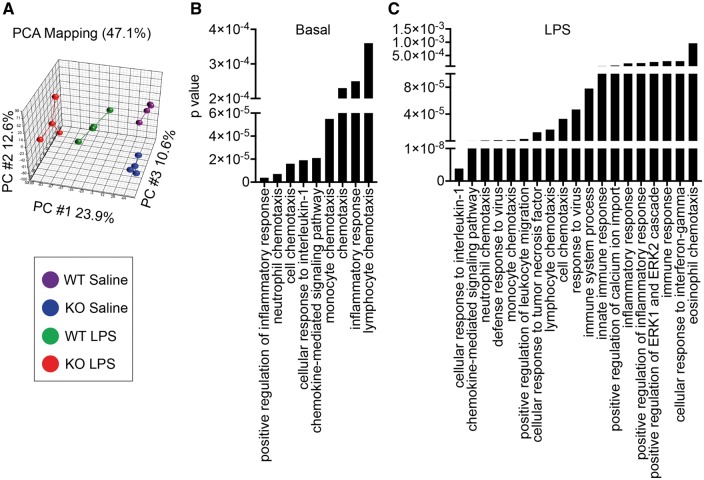
*Trem2* deficiency alters CNS transcriptomes in both basal and inflammatory conditions. RNAseq was conducted on RNA extracted from PBS perfused hippocampal tissues from male C57BL/6 N wild type (WT) or *Trem2^−/−^* (KO) mice 48 h post i.p. challenge with saline or 2 mg/kg LPS. (**A**) Principal component analysis (PCA) of RNAseq datasets of hippocampal transcriptomes for WT and KO mice treated with LPS or saline is shown. Centroids for each group are indicated on the PCA plot. *N* = 3 animals per group. WT saline, KO saline, WT LPS, and KO LPS formed four non-overlapping groups on the PCA plot, indicating they have distinct transcriptomes. Gene ontology biological process (GO BP) pathway analysis demonstrating pathways that are significantly altered in *Trem2^−/−^* (KO) mice, relative to WT in (**B**) saline injected (basal levels) and (**C**) LPS challenged animals.

**Figure 5.**
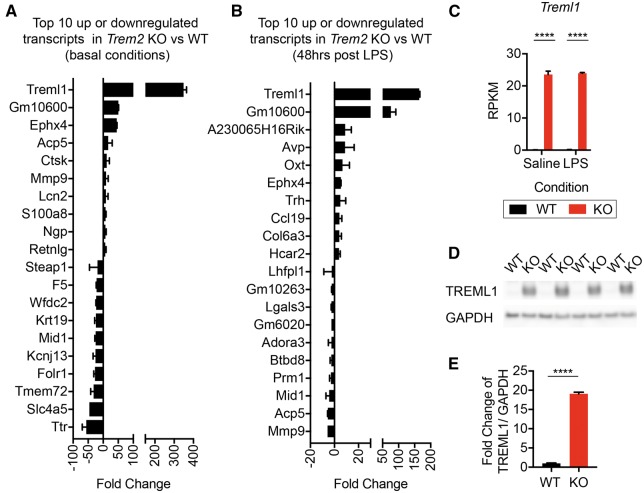
Velocigene targeted *Trem2* knockout mice have exaggerated levels of *Treml1*. The top 10 transcripts that were upregulated or downregulated in *Trem2^−/−^* relative to WT male hippocampal tissue at 48 h post i.p. injection of (**A**) saline or (**B**) 2 mg/kg LPS, as determined by RNAseq. *Treml1* was increased 348.7 ± 15.6 and 165.6 ± 1.8 in Trem*2^−/−^* over WT controls in saline and LPS injected animals, respectively. *Trem2* itself was excluded from the analysis of candidate transcripts. Shown are the averages ± S.E.M. from the *N*=3 animals per group. Data analysed by *t*-test with two-stage linear step-up procedure of Benjamini, Krieger and Yekutieli with false discovery rate (FDR) set at 5%. (**C**) The RPKM values for *Treml1*, the most abundant transcript upregulated in both saline and LPS treated *Trem2^−/−^* mice, is shown for WT (black) and *Trem2^−/−^* (red) mice. Shown are the averages ± S.E.M. from the *N*=3 animals per group. (Two-way ANOVA, Tukey post-hoc, ****P≤ 0.0001). (**D**) Western blot analysis of TREML1 and GAPDH levels in both WT and *Trem2^−/−^* mice was assessed. Representative images from *N*=4 of the *N*=10 animals per group assayed are shown. (**E**) Fold change of TREML1/GAPDH ratios normalized to the average TREML1/GAPDH ratio of WT controls are shown as the averages ± S.E.M. for *N*=10 animals per group (Unpaired two-tailed *t*-test, ****P≤ 0.0001).

Examination of the CNS transcriptome in saline and LPS treated *Trem2^−^^/^^−^* and WT mice demonstrated some alterations in the gene expression patterns, and principle component analysis (PCA) revealed four distinct populations that were separated by both genotype and LPS treatment (Fig. 4A, Supplementary Material, Fig. S1). Gene ontology analysis revealed significant alterations in pathways involving inflammation and chemotaxis of immune cells including neutrophils, monocytes, and lymphocytes as well as regulation of inflammation in *Trem2^−^^/^^−^* mice relative to WT controls under basal conditions (Fig. 4B). After peripheral LPS challenge, pathways involved in cellular responses to pro-inflammatory cytokines, including IL-1 and TNF were significantly altered (Fig. 4C). Several transcripts were altered in *Trem2^−^^/^^−^* mice relative to WT mice during basal conditions including *Treml1, Ctsk*, *Mmp9*, *Lcn2*, and *S100a8*, while many additional transcripts were altered in LPS-treated *Trem2^−^^/^^−^* mice relative to WT including *Treml1, Avp, Ccl19, Acp5*, and *Mmp9 (*Fig. 5 and Supplementary Material, Tables S1 and S2).

### *Trem2* deletion alters *Treml1* expression depending on targeting construct

Under both basal and LPS-stimulated conditions, *Treml1* was the top upregulated transcript in *Trem2^−^^/^^−^* mice, with a 349-fold change under basal conditions and a 165-fold change after LPS treatment (Fig. 5A and B and Supplementary Material, Tables S1 and S2). The deletion targeting strategy by Velocigene used a selection cassette (lacZ-flox-human Ubiquitin C promoter-neomycin-flox) to replace the coding sequence 16 bp upstream of the ATG start codon and ending at the TGA stop codon of *Trem2*. Interestingly, the *Treml1* locus is directly downstream of *Trem2*, within ∼8, 000 bp of the 3’ end of *Trem2*. Examination of the raw RPKM values in Velocigene *Trem2* targeted tissue by RNAseq demonstrated a significant increase in *Treml1* expression based solely on genotype and not LPS treatment (Fig. 5C), suggesting an artifact of the targeting strategy itself. To examine whether TREML1 protein expression was also altered, we performed a western blot on hippocampal lysates from WT or *Trem2^−^^/^^−^* mice (Fig. 5D). TREML1 levels were significantly increased in *Trem2^−^^/^^−^* mice, with the TREML1/GAPDH ratio being 19 fold higher than WT controls (1.00 ±0.09 vs 19.07 ±0.42 WT vs *Trem2^−^^/^^−^* Ave ± S.E.M.) (Fig. 5D and E).

**Figure 6.**
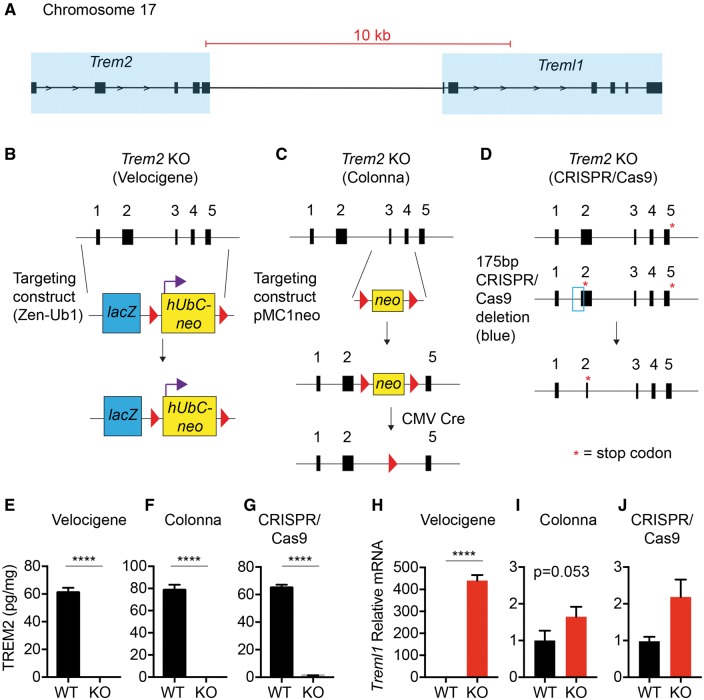
*Treml1* expression varies depending on the *Trem2* knockout strategy. (**A**) Schematic of the relationship between *Trem2* and *Treml1* on Chromosome 17. Scale bar is indicated in red, transparent blue boxes indicate regions containing exons of the respective genes, and black arrows indicate direction of transcription. *Trem2* targeting strategies for the (**B**) Velocigene, (**C**) Colonna, and (**D**) CRISPR/Cas9 approaches are depicted. TREM2 protein levels were determined by Meso Scale Discovery ELISA from Triton-X fractions of hippocampal tissues in (**E**) Velocigene, (**F**) Colonna, and (**G**) CRISPR/Cas9 derived *Trem2^−/−^* male mice, confirming that each line has no TREM2 (knockout verification). Shown are the averages ± S.E.M from *N* = 3–4 animals per group (Unpaired two-tailed *t*-test, ****P≤ 0.0001). (G–**I**) Hippocampal RNA was isolated from respective male wild-type (WT) and *Trem2^−/−^* mice (KO) for RT-qPCR analysis of *Treml1* expression in (H) *Trem2^−/−^* mice created using the Velocigene strategy and their WT C57BL/6 N controls, (I) *Trem2^−/−^* mice from the Colonna targeting, and (**J**) *Trem2^−/−^* mice from CRISPR/Cas9 targeting and controls. Shown are the averages ± S.E.M. from *N* = 6–7 animals per group. (Unpaired two-tailed *t*-test, ****P≤ 0.0001).

Examination of the genomic positioning of *Trem2* and *Treml1* revealed that the *Treml1* is located less than 10 kb downstream of *Trem2* on Chromosome 17 (Fig. 6A schematic). To determine if the disruption of the *Trem2* gene using the Velocigene targeting strategy introduced a possible artifactual upregulation of *Treml1* expression, other *Trem2* knockout lines with different targeting strategies were obtained. We compared *Trem2^−^^/^^−^* (Velocigene) mice to *Trem2^−^^/^^−^* mice from the Colonna lab [targeting that replaced exons 3 and 4 with a neomycin cassette that was subsequently removed with CMV Cre (24)], along with *Trem2^−^^/^^−^* mice from CRISPR/Cas9 targeting that created a 175 bp deletion resulting in a stop codon at amino acid 17 (Fig. 6B–D). Using a sensitive MesoScale Discovery sandwich ELISA, we measured TREM2 protein levels and found that each deletion strategy resulted in ablation of TREM2 expression relative to controls (Fig. 6E–G). To assess whether the different targeting strategies differentially impacted *Treml1* expression, RT-qPCR for *Treml1* transcript levels was conducted from hippocampal RNA. In accordance with our RNAseq data (Fig. 5C), the Velocigene targeting of *Trem2* resulted in a highly significant upregulation of *Treml1* expression with a 440.2 ± 25.26 (avg ± S.E.M.) fold induction over controls (Fig. 6H). A trend (*P* = 0.053) toward a significant increased in *Treml1* was observed in hippocampal *Trem2^−^^/^^−^* tissue derived from the Colonna deletion strategy; however, the magnitude of induction was only approximately two-fold and could be a true biological effect (Fig. 6I). Finally, although trending toward an increase, the CRISPR/Cas9 targeting of *Trem2* by Jackson Labs did not induce any significant changes in *Treml1* expression (Fig. 6J). Notably, *Ephx4* transcripts were significantly increased in both Trem2 deficient animals but with much more upregulation in the Velocigene line compared with the CRISPR/Cas9 line. Additionally, *S100a8* was only significantly altered in Velocigene animals (Supplementary Material, Fig. S2). This suggests that some, but not all, potential targets may be impacted by *Treml1* overexpression whereas others may be a result of *Trem2* deficiency itself. Together, these data indicate that these different targeting strategies have distinct outcomes on downstream *Treml1* expression.

### Deletion of the floxed neomycin cassette eliminates aberrant *Treml1* expression in Velocigene targeted *Trem2^−^^/^^−^* mice

**Figure 7.**
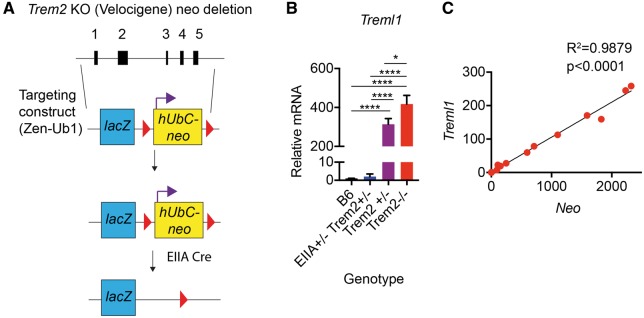
Removal of the neomycin cassette from the Velocigene *Trem2* knockout mice ameliorates aberrant *Treml1* expression. (**A**) Schematic of *Trem2^−/−^* (Velocigene) male mice crossed with EIIA-Cre mice to remove the floxed neomycin cassette driven by the human Ubiquitin C promoter. The first cross of *EIIA*-*Cre* to *Trem2^−/−^* (Velocigene) mice creates a mosaic of neomycin deletion due to some inefficiencies in Cre activity at early embryonic stages. (**B**) RT-qPCR for *Treml1* levels in hippocampal tissue from *EIIA*-*Cre^+/−^ Trem2^+/−^* mice that were negative for neomycin, were compared with WT, *Trem2^+/−^*, and *Trem2*^−/−^ Velocigene controls. *EIIA*-*Cre^+/−^ Trem2^+/−^* mice that were negative for neomycin had significantly decreased levels of *Treml1* relative to both *Trem2^+/−^*, and *Trem2^−/−^* Velocigene controls and were similar to WT. Shown are the averages ± S.E.M from *N* = 3–5 animals per group (One-way ANOVA, Tukey posthoc, *P <0.05, ****P≤ 0.0001). (C) RT-qPCR for *Treml1* and *neomycin* levels in hippocampal tissue from *EIIA Cre^+/−^ Trem2^+/−^* mice over a range of mosaicism was conducted. A highly significant, positive correlation between *Treml1* and *neomycin* expression was observed. Shown is the correlation from *N* = 18 animals ranging in *neomycin* expression. (Linear regression, *R*^2^=0.9879, *P* < 0.0001).

To examine if the human ubiquitin C promoter/neomycin cassette portion of the Velocigene *Trem2^−^^/^^−^* construct impacted transcription of the downstream *Treml1* locus, we crossed Velocigene *Trem2^−^^/^^−^* mice with *EIIA-Cre* expressing animals in order to delete the floxed selection cassette (Fig. 7A). In contrast to *Trem2*^*+*^^*/*^^*−*^ mice that retained the neo cassette, the resulting *EIIA-Cre^+/^^−^ Trem2^+/^^−^* mice, which were negative for neomycin expression, no longer expressed aberrant levels of *Treml1* (Fig. 7B). Since the *EIIA-Cre* mice results in different levels of mosaicism in *neomycin* deletion after the first cross (prior to establishing a germline colony), we were able to examine whether there was a correlation between *neomycin* and *Treml1* expression. Hippocampal RT-qPCR revealed a strong positive correlation between *neomycin* and *Treml1* expression with an R^2^ value of 0.9879 (*P* < 0.0001) (Fig. 7C). Resolution of *Treml1* levels to wild-type levels through the excision of the human ubiquitin C promoter/neo cassette suggests that these residual elements in the Velocigene targeting construct are able to drive expression of downstream genes in the endogenous locus.

## Discussion

Microglial activation is observed in numerous neuropathologies, from neurodegeneration to autoimmune disease, and may contribute to disease through the production of pro-inflammatory mediators (5,6). As a result, regulation of the progression and resolution of microglial responses can be critical to the outcome of disease. Several cell surface regulatory molecules on microglia, including CX3CR1 and CD200R1, are thought to modulate function (47,48). Although TREM2 is almost exclusively expressed on microglia and TREM2 variants are associated with neurological disease, the impact of TREM2 on CNS function is still under debate. Overall, we have demonstrated that the targeting strategy used to study the role of TREM2 on CNS sequelae in mouse models is pertinent in the interpretation of data. Using the Velocigene *Trem2^−^^/^^−^* mice, we have demonstrated that TREM2 does not impact basal behavioral or cognitive readouts in adult animals or result in large shifts in hippocampal ISF early inflammation following systemic LPS challenge. As TREM2 has been shown to impact microglial expansion during aging and has been shown to impact survival in both macrophages and microglia, it is possible that behavioral and cognitive readouts would be different in an aged cohort of *Trem2^−^^/^^−^* mice (44,45,49). Importantly, RNAseq analysis of CNS transcriptomes revealed exaggerated *Treml1* expression that was over 300-fold in *Trem2^−^^/^^−^* (Velocigene) mice relative to controls during basal conditions. However, this large elevation in *Treml1* expression was not observed in brain tissues derived from other *Trem2* KO mice using two independent targeting strategies. Additionally, removal of the floxed neomycin cassette driven by the human ubiquitin C promoter in the Velocigene targeted *Trem2* line completely prevented the *Treml1* overexpression artifact, indicating that the promoter activity was sufficient to drive expression of a locus over 8 kb downstream. Because many Velocigene targeting strategies utilize this selection cassette, we suggest removal of the floxed neomycin cassette in order to circumvent transcription of neighboring genes. Due to this confound, it is therefore uncertain as to whether *Treml1* is either compensating for or masking the impact of TREM2 deficiencies in these animals.

Although it is unknown if and how *Treml1* impacts the overall response in *Trem2* deficient animals, our RNAseq data demonstrated alterations in inflammatory pathways in the absence of *Trem2*. During basal conditions, increased levels of *Ephx4*, *Mmp9*, *Ctsk*, and *S100a8* were within the top 10 induced genes. *S100a8* and *S100a9*, the 11^th^ most upregulated transcript in *Trem2^−^^/^^−^* mice in our data set, have been shown to play a role focal cerebral ischemia, with a deficiency in both transcripts resulting in diminished disease (50). Epoxide hydrolases are also involved in the regulation of neuroinflammation and inhibition of soluble epoxide hydrolases resulted in decreased pro-inflammatory cytokines (IL-1β and Il-6) within the context of seizures, and increased IL-10 secretion in the CNS following cardiac arrest (51,52). Additionally, both *Mmp9* and *Ctsk* are also associated with microglial activation (53). *Trem2^−^^/^^−^* mice also show a significant upregulation of *Lcn2*, which we been shown to be a potent anti-inflammatory molecule in the context of peripheral LPS challenge (54). These changes may reflect a compensatory mechanism to offset the upregulation of the aforementioned transcripts that are likely to be more pro-inflammatory. Therefore, these data suggest that TREM2 may be playing an anti-inflammatory role in the CNS.

Recently, the role of TREM2 on microglial function has been under intense investigation in light of data associating TREM2 variants with an increased AD risk (18–20,55,56). Several studies using *Trem2^−^^/^^−^* mice from Velocigene targeting or Colonna lab targeting have revealed alterations in the clustering and morphology of plaque associated myeloid or microglial cells (34,46,57). However, there are conflicting results that remain regarding not only in the origin of the plaque-associated cells (i.e. peripherally derived TREM2^+^ myeloid cells vs TREM2^+^ microglia) but the role of TREM2 in amyloid pathology. Usage of the Velocigene *Trem2^−^^/^^−^* mice demonstrated slightly decreased amyloid pathology whereas a study using the Colonna derived *Trem2^−^^/^^−^* mice showed a slight increase in pathology in the hippocampus (34,57). Recently, it has been suggested that TREM2 functions in a distinct manner based on the stage of AD pathology, with an ability to mitigate early disease and exacerbate late disease (58). However, given our findings that there is a large discrepancy in the expression of *Treml1* between the two model systems, this may play a significant role in shaping disease. Interestingly, *TREML1* levels in the brain have recently been associated with decreased AD risk in humans (59), suggesting that upregulated expression may be protective in disease. Although soluble TREML1 can decrease LPS induced neutrophil inflammation suggesting an anti-inflammatory role with that cell type, to our knowledge nothing is currently known regarding the role of TREML1 and microglial function (60). Whether exaggerated *Treml1*, which is observed in the Velocigene *Trem2^−^^/^^−^* mice, is also playing a protective role when crossed to an amyloid model of disease is unknown. Therefore, caution must be taken when using the Velocigene *Trem2^−^^/^^−^* mice to define the role of TREM2 on microglial function until more is known regarding the nature of TREML1.

## Materials and Methods

### Animals

C57BL/6 N mice, *Trem2^−^^/^^−^* mice (KOMP line #10093 generated by Velocigene), and Trem2^CRISPR-KO^ mice (Jackson Labs stock #027197) were housed under standard laboratory conditions in ventilated cages on 12-h light: dark cycles in a specific pathogen-free environment at Mayo Clinic. *Trem2^−^^/^^−^* mice generated by the Colonna lab (24) were also housed under standard laboratory conditions in ventilated cages on 12-h light: dark cycles in a specific pathogen-free environment. For LPS studies, animals were injected intraperitoneally (i.p.) with 2 mg/kg LPS (0111: B4; Sigma, Saint Louis, MO). Animal protocols were reviewed and approved by Mayo Clinic Institutional Animal Care and Use Committee.

### Behavioral battery

To measure readouts of anxiety, mobility, sociability, and cognition, a behavioral battery consisting of open field assay (OFA), elevated plus maze (EPM), three chambered social interaction (3SI), and contextual and cued fear conditioning was performed on consecutive days, as previously described (61,62). All testing was conducted between 9: 00 A.M. and 5: 00 P.M. and animals were acclimated to the testing room for 1 h prior to the onset of testing. Prior to the initial test and in between each test animal, the behavioral apparatus was cleaned with 30% ethanol (or 30% isopropanol for cued fear conditioning) to remove any residual odors on the equipment.

### Open field assay

To assess activity/anxiety in the open field assay (OFA), mice were placed in a 40x40x30 cm (WxLxH) black Plexiglass box with a brightly lit center. Fujinon overhead cameras recorded behavioral activity for 15 min and ANY-maze software was utilized for tracking (Stoelting Co.; Wood Dale, IL). An imaginary 13  × 13 cm center region in the box was digitally defined using ANY-maze software. Side mounted photobeams located 7.6 cm from the floor of the box were used to monitor rearing activity. Multiple readouts including total distance traveled, time mobile, center: total distance, and rearing were analysed.

### Elevated plus maze

Elevated plus maze (EPM) is another test for assessing anxiety-like behavior. A four- armed (50 × 10 cm) maze is elevated 50 cm above the floor, with two of the arms enclosed by open top gray walls (35 × 15 cm) while the other two remain ‘open’. Mice were placed in the maze in the same orientation and their exploratory behavior was monitored for 5 min using an overheard camera and ANY-maze software. The ratio of time spent in open: closed arms was utilized as an anxiety measurement.

### Three-chambered social interaction

To examine sociability, mice were placed in a three-chamber Plexiglass 40×40 cm box consisting of two 17 × 40 cm regions separated by 2 dividers forming a smaller 5×40 cm center region. Mice were able to move freely through a small 8×5 cm opening that was aligned in both dividers. Two mesh cylindrical containers were placed in the box, one in the corner of the ‘empty’ chamber and one in the corner of the ‘mouse’ chamber. The test mouse was placed in the box for 4 min to explore the surroundings and then removed and placed in a temporary holding cage. A same sex matched probe mouse was placed under the mesh container in the ‘mouse’ chamber compartment allowing for visual, olfactory, and auditory interactions between probe and test mice. After placement of the probe mouse, the test mouse was returned and allowed to explore the chambers undisturbed for 10 min. All behavior was tracked with and overhead camera and ANY-maze software. The interaction score was calculated as: (time spent near the mouse container- time spent near the empty container)/(time spent near the mouse container + time spent near the empty container).

### Context and cued fear conditioning

To measure freezing as a learning/memory readout, mice were trained in a sound attenuated chamber with an overhead mounted camera and FreezeFrame tracking software (Actimetrics, Wilmette, IL) and a floor consisting of rods that were capable of inducing a mild electrical stimulus. Initial training of mice occurred by allowing animals to remain undisturbed for 2 min in the chamber while baseline freezing behavior was recorded. This was followed by a 30 s conditioned stimulus (CS) of an 80 dB white noise that was paired with an unconditioned stimulus (UC) of a mild foot shock (0.5 mA) for the final 2 s of the CS. Mice were undisturbed for the next consecutive minute and then received a second CS-US session followed by another 30 s of rest. Afterward, mice were removed from the chamber, returned to their home cage and left undisturbed overnight. The following day, mice were re-acclimated to the behavior room under the same conditions, returned to the test chamber, and freezing behavior was recorded for 5 min to examine contextual learning/memory (context test). Mice were then returned to their home cage, moved to a different room with dimmed lighting, and allowed to re-acclimate to these new conditions for a minimum of 1 h. To examine cued learning/memory (cued test) to the auditory CS independent of the previous contextual cues, several parameters of the environment were altered including both visual (i.e. changing from white to red ceiling lighting, dimming the anterior chamber, altering the test chamber itself by covering the floor with a white opaque plastic, changing the shape and spatial cues within the chamber, and altering the cages used to transport the animals to the test chamber) and olfactory (i.e. replacing 30% ethanol with 30% isopropanol to wipe down the test chamber, adding a vanilla extract scent to the test chamber and the anterior room). Mice were placed in the altered chamber for 3 min followed by initiation of the auditory CS for 3 min during which time freezing behavior was recorded. For both context and cued tests, baseline-freezing behavior obtained during the training period was subtracted from the overall freezing score to control for animal variability.

### Tissue processing for RNAseq and RT-qPCR

Mice were deeply anesthetized with pentobarbital prior to cardiac perfusion with phosphate-buffered saline (PBS) to expunge blood components from the cerebrovasculature. For biochemical analysis, dissected hippocampal brain tissues were quickly frozen on dry ice and stored at −80 °C until further processing. Tissues were sonicated with 2–3 pulses in Tris buffered saline with EDTA (TBSE) (50 mM Tris pH 7.5, 150 mM NaCl, 1 mM EDTA) with 1x protease and phosphatase inhibitors (Thermo Scientific, Waltham, MA). An aliquot of the tissue suspension was processed for RNA isolation using either the Aurum Total RNA mini kit (Biorad, Hercules, CA) or RNeasy Plus kit (Qiagen, Valencia, CA). Random-primed reverse transcription was performed according to manufacturer protocols (Invitrogen-Life Technologies, Grand Island, NY). cDNA was added to a reaction mix (10 μl final volume) containing 300 nM gene-specific primers and Universal SYBR green supermix (Biorad, Hercules, CA). All samples were run in triplicate and were analysed on a Quant Studio 7 Flex Real Time PCR instrument (Applied Biosystems - Life Technologies). Relative gene expression was normalized to GAPDH controls and assessed using the 2^-ΔΔCT^ method. Primer sequences are as follows (5’ to 3’): *Gapdh* F: CTGCACCACCAACTGCTTAG, *Gapdh* R: ACAGTCTTCTGGGTGGCA GT, *Aif1*(Iba1) F: GGATTTGCAGGGAGGAAAAG, *Aif1*(Iba1) R: TGGGATCATCGAGGAATTG, *Treml1* F: GTGATGGCCAGAAAGAAAGG, *Treml1* R: TTCCTGGAACTCCAGTGCTC, *Neo* F: TGAATGAACTGCAGGACGAG, *Neo* R: AGTGACAACGTCGAGCACAG, *Ephx4* F: TCATTGTTATTAACTTCCCACATC, *Ephx4* R: AACAGCTGGGCAGGATGC, *S100a8* F: CCTTTGTCAGCTCCGTCTTC, *S100a8* R: CAAGGCCTTCTCCAGTTCAG

### Meso-scale discovery ELISA

PBS perfused hippocampal brain tissues were isolated and briefly sonicated with 2–3 pulses in Tris buffered saline with EDTA (TBSE) (50 mM Tris pH 7.5, 150 mM NaCl, 1 mM EDTA) with 1x protease and phosphatase inhibitors (Thermo Scientific, Waltham, MA). An aliquot was added to an equal amount of 2% Triton TBSE with 1x protease and phosphatase inhibitors for a final concentration of 1% Triton. Samples were lysed for 30 min on ice and then centrifuged at 14, 000 *g* for 15 min. Triton-X supernatants were stored at −80 °C until further use. For MSD ELISA, 96 well plates were coated with streptavidin (Meso Scale Discovery, Rockville, MD) and then washed and blocked in 0.5% BSA/PBST (0.05% Tween-20) overnight at 4 °C. Plates were incubated with 0.25 μg/ml biotinylated sheep anti-mouse TREM2 antibody (R&D Systems, Minneapolis, MN) for 1 h at RT. Following three washes with PBST, samples and standards diluted in 0.25% BSA/PBST supplemented with protease inhibitors (Roche, Indianapolis, IN) were incubated overnight at 4 °C. Plates were washed three times with PBST and incubated with 1 μg/ml rat monoclonal anti-mouse TREM2 (R&D Systems, Minneapolis, MN) for 1 h at RT. After 4 washes in PBST, 0.5µg/ml SULFO-TAG labeled anti-rat antibody was added and incubated for 1 h protected from light. Plates were washed 3 times in PBST and then once in PBS prior to being developed in Meso Scale Discovery Read buffer and read on the Meso Scale Discovery SECTOR Imager 2400 (Meso Scale Discovery, Rockville, MD).

### Western blot

Hippocampal tissues from PBS perfused mice were sonicated with 2–3 pulses in Tris buffered saline with EDTA (TBSE) (50 mM Tris pH 7.5, 150 mM NaCl, 1 mM EDTA) with 1x protease and phosphatase inhibitors (Thermo Scientific, Waltham, MA). An aliquot of the sample was incuvated with 2% Triton-X TBSE with 1x protease and phosphatase inhibitors for a final concentration of 1% Triton-X TBSE (TBSE-X). Samples were incubated for 1 h on ice and the centrifuged at 20, 000rcf at 4 °C for 15 min. Supernatants were examined for protein concentration using a BCA assay (ThermoScientific, Waltham, MA). 15 μg of protein was boiled for 5 min and resolved on a 10% polyacrylamide bis-tris gel and transferred to a nitrocellulose membrane. Membranes were blocked in 5% non-fat dry milk in PBS with 0.1% Tween 20 (PBS-T) (Sigma) and probed overnight at 4 °C with 1: 500 biotinylated goat anti-TREML1 (R&D Systems, Minneapolis, MN), followed by horseradish peroxidase (HRP) conjugated mouse anti-biotin secondary antibody. Membranes developed using supersignal west dura extended duration chemiluminescent substrate (Thermo Scientific) prior to film exposure. Membranes were stripped and re-probed with 1: 5000 mouse anti-GAPDH (clone 6C5, Advanced Immunochemicals, Long Beach, CA) overnight 4 °C, followed by goat anti-mouse HRP (Jackson Immunoresearch Laboratories). Membranes were washed and developed with enhanced chemiluminescent substrate (Thermo Scientific).

### Immunohistochemistry

PBS perfused hemi-brains were drop fixed into 10% neutral buffered formalin (Fisher Scientific, Waltham, MA) overnight at 4 °C. Tissue was then transferred to 30% sucrose in PBS overnight at 4 °C. Coronal brain sections (50 μ) were cut on a freezing-sliding microtome and stored at −20 °C in cryprotectant until staining. Sections were washed in PBS to remove cryoprotectant and then blocked for endogenous peroxidase activity and permeabilized with 0.6% H_2_O_2_, 0.1% NaN_3_ in PBS-X (1X PBS containing 0.3% Triton-X) for 30 min at RT. Sections were blocked with 1% milk in PBS-X followed by incubation with rabbit anti-Iba1 at 1: 6000 (cat# 019–9741, Wako, Richmond, VA) in 0.5% milk PBS-X for 2 days at 4 °C. Sections were then incubated with the Vectastain anti-Rabbit IgG kit (Vector Labs, Burlingame, CA) overnight at 4 °C followed by ABC component for 4 h and developed using the DAB kit (Vector Labs) according to manufacturer’s instructions. Images were acquired using an Aperio XT Scanner (Aperio, Vista, CA) at a 20x magnification.

### *In vivo* microdialysis

*In vivo* microdialysis of freely moving, awake mice was performed as described (63). Mice were provided acetaminophen 48 h before surgical probe implantation. Mice were anesthetized using 1.5–2.5% isoflurane prior to shaving the head and transecting the skin along the midline to expose the skull. A bore hole (0.75 mm) was made above the left hippocampus (coordinates bregma -3.1 mm, 2.5 mm lateral) using a small animal sterotaxic device. A second bore hole was made into the right, posterior skull quadrant for an anchoring bone screw. An AtmosL Guide Cannula (PEG-X, Eicom) was then stereotactically inserted into the hippocampus (12° angle, dura mater -1.2 mm) and cemented into place with dental cement. An AtmosLM Dummy probe (PED-X, Eicom) was inserted into the guide cannula and the wound was closed with surgical adhesive glue. Body temperature was maintained during the procedure using a heating blanket set at 37 °C. Mice were injected with antibiotic (ampicillin; 100 mg/kg, i.m.) and placed into a clean cage and provided with access to food and water ad libitum. Mice were allowed to awaken and recover overnight. To sample large molecules from the extracellular space, microdialysis MegaProbes (1000-kDa MWCO membrane, Eicom) were used with a peristaltic push-pull pump (SciPro). Microdialysis perfusion buffer was aCSF (1.3 mM CaCl_2_, 1.2 mM MgSO_4_, 3 mM KCl, 0.4 mM KH_2_PO_4_, 25 mM NaHCO_3_, 122 mM NaCl, pH 7.35) containing 0.15% BSA (Sigma) that was filtered through a 0.1 μM membrane. Mice were kept under constant light conditions for the remainder of the experiment. LPS (2 mg/kg) was injected (i.p.) 10 h post probe insertion. Microdialysis was performed at a flow rate of 1 μl/min and interstitial fluid (ISF) samples were collected hourly using a refrigerated fraction collector (SciPro).

### Cytometric bead array

ISF samples were pooled into 2-h fractions and assayed for pro-inflammatory cytokines using a mouse inflammatory cytokine bead array kit according to manufacturer’s instructions (BD Bioscience, San Jose, CA). Samples were collected on a FACS Calibur (BD Bioscience, San Jose, CA) and analysed with FlowJo software (Tree Star, Inc).

### Illumina RNA sequencing and pathway analysis

A total of 12 mRNA samples were sequenced at Mayo Clinic Genome Facility using NuGen Ovation library preparation and sequenced on an Illumina HiSeq 4000. Reads were mapped to the mouse genome mm10 and reads per kilobase per million mapped reads (RPKM) were generated for each transcript. RPKM values were log2 transformed and low abundance transcripts were removed from further analysis (log2 RPKM value less than -2 across all experimental groups were considered too lowly expressed). Principal component analysis (PCA) was performed using Partek Genomics Suite (Partek Inc., St. Louis, MO) with ANOVA and multiple comparison adjustment of p values using *Benjamini–Hochberg-Yekutieli* correction with false discovery rate (FDR) set at 5%.

## Supplementary Material

Supplementary Material is available at *HMG* online.

*Conflict of Interest statement.* None declared. 

## Funding

Mayo Foundation, GHR Foundation, Mayo Clinic Center for Individualized Medicine, Mayo Clinic Gerstner Family Career Development Award, Ed and Ethel Moore Alzheimer’s Disease Research Program of Florida Department of Health (6AZ06), NIH NS094137, AG047327 and AG049992 (JDF), The Robert and Clarice Smith and Abigail Van Buren Alzheimer’s Disease Research Program Fellowship, Mayo Clinic Program on Synaptic Biology and Memory, and NIH MH103632 (SK), and NIH AG027924, AG035355, and NS074969 (GB). Funding to pay the Open Access publication charges for this article was provided by the Mayo Foundation.

## Supplementary Material

Supplementary Fig legend

Supplementary Figure 1

Supplementary Figure 2

Supplementary Table I

Supplementary Table II
