# Supplementary material for: Behavioral and transcriptomic analysis of Trem2-null mice: not all knockout mice are created equal
Source: Hum Mol Genet. 2017 Oct 11;27(2):211–23. doi: 10.1093/hmg/ddx366 (PMC5886290; doi:10.1093/hmg/ddx366)
Supplement: Supplementary Fig legend [file ddx366_hmg-2017-twb-00677.r1_kang_supplementary_fig_legend.doc]

**Supplementary Figure 1.**

RNAseq conducted from PBS perfused hippocampal tissues from C57BL/6N wild type (WT) or *Trem2^-/-^* (KO) mice 48 hours post i.p. challenge with saline or 2μg/g LPS. Hierarchical clustering of RNAseq datasets of hippocampal transcriptomes for WT and KO mice treated with LPS or saline is shown for WT saline (green), KO saline (orange), WT LPS (yellow), and KO LPS (red). N=3 mice per group.

**Supplementary Figure 2.**

RT-qPCR conducted on PBS perfused hippocampal tissues from C57BL/6N wild type (WT) or *Trem2^-/-^* (KO) Velocigene mice or CRISPR/Cas9 targeted Trem2 targeted KO mice and WT controls were examined for (A) *Ephx4* or (B) *S100a8* expression. Shown are the averages ± S.E.M. from N=6 animals per group for the Velocigene and controls and N=10-12 WT vs CRISPR/Cas9 mice. (Unpaired two-tailed t-test, ****p≤ 0.0001. **p<0.01, *p<0.05)
