# Supplementary figures and images for: Behavioral and transcriptomic analysis of Trem2-null mice: not all knockout mice are created equal
Source: Hum Mol Genet. 2017 Oct 11;27(2):211–23. doi: 10.1093/hmg/ddx366 (PMC5886290; doi:10.1093/hmg/ddx366)

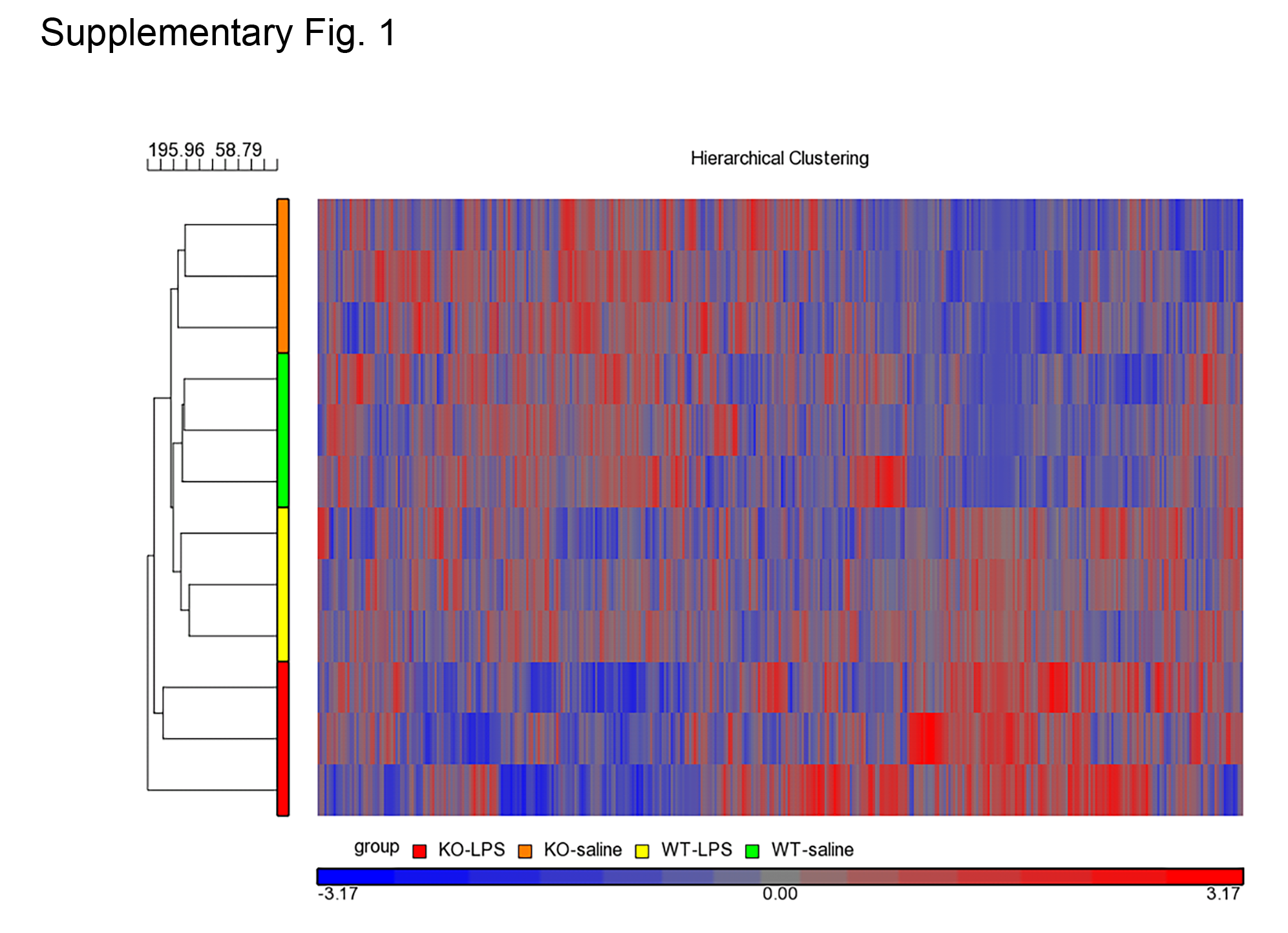

Supplement: Supplementary Figure 1 [file ddx366_hmg-2017-twb-00677_kang_supplementary_figure_1.png]

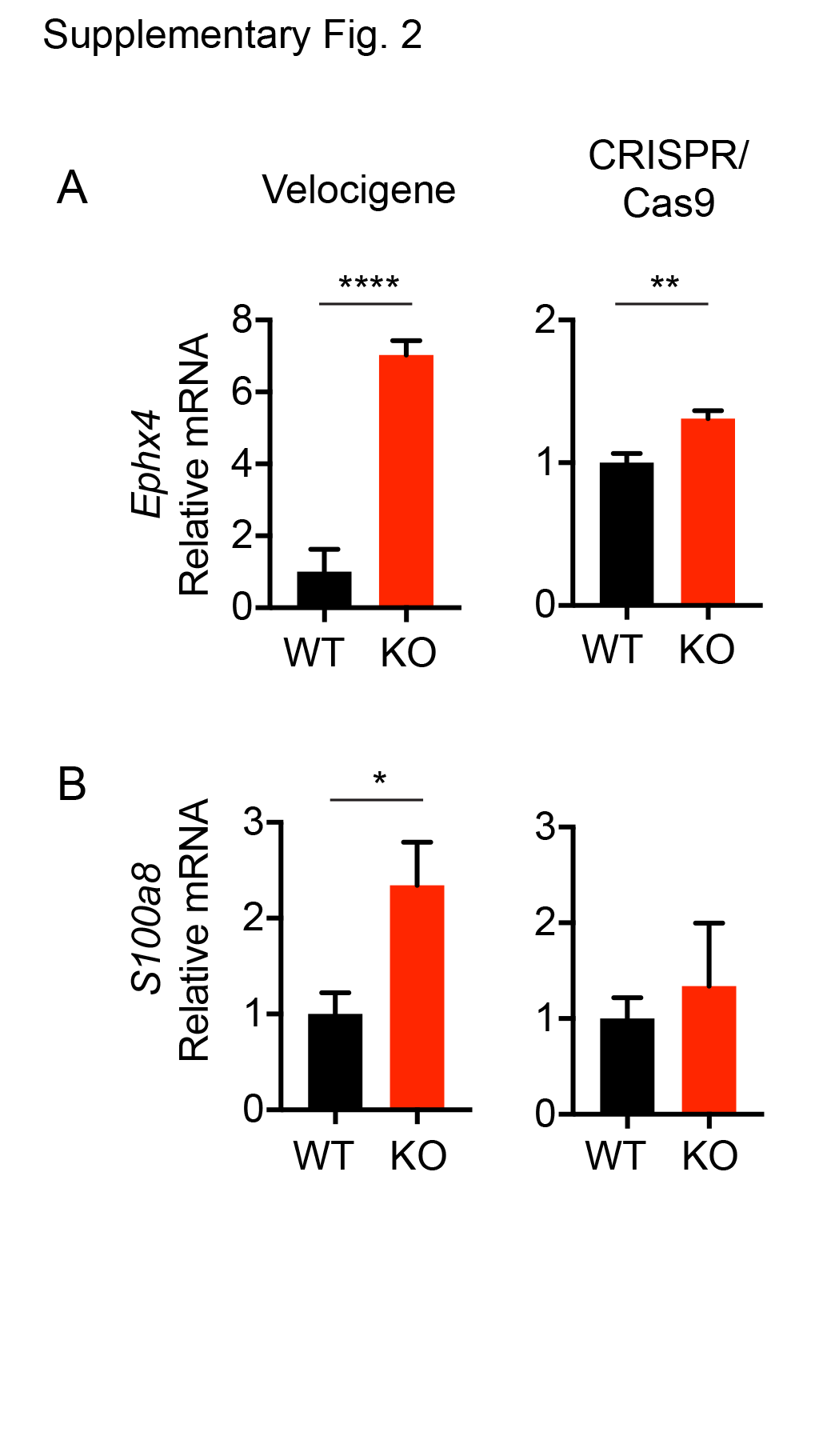

Supplement: Supplementary Figure 2 [file ddx366_hmg-2017-twb-00677_kang_supplementary_figure_2.png]
